# Mosaic trisomy of chromosome 1q in human brain tissue associates with unilateral polymicrogyria, very early-onset focal epilepsy, and severe developmental delay

**DOI:** 10.1007/s00401-020-02228-5

**Published:** 2020-09-26

**Authors:** Katja Kobow, Samir Jabari, Tom Pieper, Manfred Kudernatsch, Tilman Polster, Friedrich G. Woermann, Thilo Kalbhenn, Hajo Hamer, Karl Rössler, Angelika Mühlebner, Wim G. M. Spliet, Martha Feucht, Yanghao Hou, Damian Stichel, Andrey Korshunov, Felix Sahm, Roland Coras, Ingmar Blümcke, Andreas von Deimling

**Affiliations:** 1 Department of Neuropathology, Institute of Neuropathology, Affiliated Partner of the ERN EpiCARE, Universitätsklinikum Erlangen, Friedrich-Alexander-University of Erlangen-Nürnberg (FAU), Schwabachanlage 6, 91054 Erlangen, Germany; 2 Department of Neurology, Schön Klinik Vogtareuth, Vogtareuth, Germany; 3 Department of Neurosurgery and Epilepsy Surgery, Schön Klinik Vogtareuth, Vogtareuth, Germany; 4 Research Institute “Rehabilitation, Transition, Palliation”, PMU Salzburg, Salzburg, Austria; 5 Epilepsy Center Bethel, Krankenhaus Mara, Bielefeld, Germany; 6 Department of Neurosurgery, Evangelisches Klinikum Bethel, Bielefeld, Germany; 7 Department of Neurology, Epilepsy Center, Universitätsklinikum Erlangen, Friedrich-Alexander-University of Erlangen-Nürnberg (FAU), Erlangen, Germany; 8 Department of Neurosurgery, Universitätsklinikum Erlangen, Friedrich-Alexander-University of Erlangen-Nürnberg (FAU), Erlangen, Germany; 9 Department of Neurosurgery, Medical University of Vienna, Vienna, Austria; 10 Department of (Neuro)Pathology, Amsterdam Neuroscience, Amsterdam UMC, University of Amsterdam, Amsterdam, The Netherlands; 11 Department of Pathology, University Medical Center Utrecht, Utrecht, The Netherlands; 12 Department of Pediatrics and Adolescent Medicine, Affiliated Partner of the ERN EpiCARE, Medical University Vienna, Vienna, Austria; 13 Department of Neuropathology, Universitätsklinikum Heidelberg, and, CCU Neuropathology, German Cancer Research Center (DKFZ), Heidelberg, Germany

**Keywords:** Chromosome 1, Copy number variation, Brain development, Cortical malformation, ID, Seizures

## Abstract

Polymicrogyria (PMG) is a developmental cortical malformation characterized by an excess of small and frustrane gyration and abnormal cortical lamination. PMG frequently associates with seizures. The molecular pathomechanisms underlying PMG development are not yet understood. About 40 genes have been associated with PMG, and small copy number variations have also been described in selected patients. We recently provided evidence that epilepsy-associated structural brain lesions can be classified based on genomic DNA methylation patterns. Here, we analyzed 26 PMG patients employing array-based DNA methylation profiling on formalin-fixed paraffin-embedded material. A series of 62 well-characterized non-PMG cortical malformations (focal cortical dysplasia type 2a/b and hemimegalencephaly), temporal lobe epilepsy, and non-epilepsy autopsy controls was used as reference cohort. Unsupervised dimensionality reduction and hierarchical cluster analysis of DNA methylation profiles showed that PMG formed a distinct DNA methylation class. Copy number profiling from DNA methylation data identified a uniform duplication spanning the entire long arm of chromosome 1 in 7 out of 26 PMG patients, which was verified by additional fluorescence in situ hybridization analysis. In respective cases, about 50% of nuclei in the center of the PMG lesion were 1q triploid. No chromosomal imbalance was seen in adjacent, architecturally normal-appearing tissue indicating mosaicism. Clinically, PMG 1q patients presented with a unilateral frontal or hemispheric PMG without hemimegalencephaly, a severe form of intractable epilepsy with seizure onset in the first months of life, and severe developmental delay. Our results show that PMG can be classified among other structural brain lesions according to their DNA methylation profile. One subset of PMG with distinct clinical features exhibits a duplication of chromosomal arm 1q.

**Electronic supplementary material:**

The online version of this article (10.1007/s00401-020-02228-5) contains supplementary material, which is available to authorized users.

## Introduction

PMG is a malformation of cortical development (MCD) characterized by an excessive number of abnormally small and partly fused, and so-called frustrane gyration together with abnormal cortical lamination [8]. PMG can be limited to a single gyrus, involving only part of one hemisphere, be bilateral and asymmetrical, bilateral and symmetrical, or diffuse [7, 55]. Clinical signs and symptoms are heterogeneous depending on how many and which brain regions are affected. They may range from mild intellectual disability, mobility and language problems to severe encephalopathy with intractable epilepsy. While PMG most often occurs as an isolated malformation, it can accompany several other brain malformations, e.g., microcephaly, megalencephaly, periventricular nodular heterotopia, FCD, agenesis of the corpus callosum and brainstem, or cerebellar abnormalities [62]. PMG is genetically heterogeneous with about 40 associated genes [34, 55, 65]. Small copy number variants (CNVs) have also been associated with PMG, but only deletions in 1p36.3 and 22q11.2 are common; otherwise, CNVs are rare [23, 57]. A causal gene has not been identified for any of the CNV loci. Among non-genetic causes of PMG hypoxia-ischemia, trauma or congenital infections mainly from cytomegalovirus have been reported [9, 39].

Microscopically, in PMG, the gyri are atypically organized with abnormalities of the physiological laminar cytoarchitectural structure, which may be unlayered or four-layered. In unlayered PMG, the molecular layer is continuous and does not follow convolutional profiles. Neurons have a radial distribution without any laminar organization. By contrast, the four-layered PMG shows a laminar structure composed of a molecular layer, an outer neuronal layer, a nerve fiber layer, and an inner neuronal layer. Occasionally, in the neuronal cell layers, granular as well as pyramidal neurons can be observed, resembling residuals of the normal six-layered cortical architecture [26]. The two histopathological PMG subtypes do not necessarily have a distinct origin, as both may coexist in contiguous cortical regions [10].

Though PMG is frequently associated with epilepsy, mechanisms of epileptogenesis related to PMG are not fully understood. Experimental data obtained in the rat freeze lesion model indicate that functional abnormalities extend beyond the anatomical malformation [33, 42]. This corroborates observations in humans, revealing that the cortex surrounding the PMG is also involved in epileptogenesis.

Classification of MCD including PMG remains challenging in everyday clinical practice [43]. DNA methylation profiling can be used for molecular classification of seemingly morphological homogenous entities. It can further provide certain information on chromosomal imbalances, and inform about molecular pathways underlying disease development. This has been proven to variable extent in brain tumors and major subtypes of focal cortical dysplasia (FCD) [35, 38, 54, 58]. We hypothesized that the determination of DNA methylation signatures might help to distinguish PMG from other related hemispheric and focal MCD and identify PMG subtypes with specific molecular and clinical features. In the present study, we, therefore, used llumina DNA Methylation 850K BeadChip Arrays to molecularly characterize 26 PMG and correlate molecular-genetic data with histomorphological and clinical phenotype.

## Materials and methods

### Study subjects

We obtained written informed consent for molecular-genetic investigations and publication of the results for all participating patients. The Ethics Committee of the Medical Faculty of the Friedrich-Alexander-University (FAU) Erlangen-Nürnberg, Germany, approved this study within the framework of the EU project "DESIRE" (FP7, grant agreement 602531; AZ 92_14B). We reviewed clinical, imaging and histological data of individuals who underwent surgery for the treatment of their focal pharmacoresistent epilepsy and were diagnosed with unilateral PMG (*n* = 26; mean age at surgery ± SEM = 12.5 ± 3.5 years; Table 1), hemimegalencephaly (HME, *n* = 6; mean age ± SEM = 1.3 ± 0.2 years), FCD type 2 (*n* = 36; mean age ± SEM = 15.8 ± 2.2 years), or temporal lobe epilepsy (TLE, *n* = 15; mean age ± SEM = 37.0 ± 4.0 years; All TLE patients had a histopathological diagnosis of hippocampal sclerosis, but only apparently normal temporal neocortex was used in the present analysis.). All disease diagnosis was based on MRI and histology. Five non-epilepsy autopsy control cases with no known neurological history were also included in the study (mean age ± SEM = 30.4 ± 8.7 years; Supplement Table 1, online resource).

**Table 1:** Clinical summary of PMG cases

#	Pathology	Additional features	Lobe	Side	Sex	Age	Onset	Duration	Outcome	Development	CNP	Other
1	Focal PMG		Frontal	L	f	3	0	3	4a	Severe delay	1q gain	
2	Hemispheric PMG		Frontal	R	m	2	1	1	1b	Severe delay	1q gain	
3	Focal PMG		Frontal	L	m	1	0	1	1a	Severe delay	1q gain	
4	Hemispheric PMG	HET	Frontal	L	m	3	0	3	1a	Severe delay	1q gain	
5	Focal PMG		Frontal	R	f	1	0	1	1a	Mild delay	1q gain	
6	Focal PMG		Frontal	R	m	15	0	15	3a	Severe delay	1q gain	
7	Hemispheric PMG	HET	Frontal	R	f	7	0	7	1a	Severe delay	1q gain	
8	Hemispheric PMG		Frontal	L	m	8	3	6	2b	Severe delay	neg	22q11del
9	Complex MCD	HME	Frontal	R	m	2	1	2	1a	Severe delay	neg	
10	Focal PMG		Frontal	R	m	1	1	0	N/A	Normal	neg	
11	Focal PMG	MTS, SWS, cerebellar lesion	Temporal	L	f	17	11	6	1a	Severe delay	neg	
12	Focal PMG	calcifications, SWS	Frontal	N/A	m	6	1	5	N/A	N/A	neg	
13	Complex MCD	HME, HET, schizencephaly	Frontal	L	m	3	1	2	1a	Severe delay	neg	
14	Complex MCD	HME, FCD2B, HET	Temporal	R	f	2	0	2	1a	Severe delay	neg	
15	Complex MCD	HME, FCD2B, HET	Temporal	L	f	2	0	2	ex.let	Severe delay	neg	
16	Complex MCD	HME, HET	Temporal	L	m	1	0	1	4	Severe delay	neg	
17	Hemispheric PMG	HET	Parietal	R	m	8	1	7	1a	Mild-to-moderate delay	neg	
18	Complex MCD	HME, FCD2A, HET	Frontal	R	m	17	1	16	1a	Severe delay	neg	
19	Complex MCD	HME, FCD2A	Temporal	R	f	5	0	5	1a	moderate delay	neg	
20	Focal PMG	HET	Frontal	R	m	35	10	25	1a	Normal	neg	
21	Focal PMG		Temporal	L	m	4	3	1	1a	Severe delay	neg	CMV
22	Hemispheric PMG		Frontal	R	m	12	3	9	1a	Severe delay	neg	
23	Hemispheric PMG		Temporal	R	m	7	2	5	1a	Severe delay	neg	
24	Hemispheric PMG		Frontal	R	m	9	3	6	N/A	Severe delay	neg	
25	Hemispheric PMG		Frontal	R	f	8	5	4	1a	Mild delay	neg	
26	Hemispheric PMG	Microcephaly	Frontal	R	m	2	1	1	1a	Severe delay	neg	

*CMV* cytomegalovirus, *CNP* copy number profile, *del* deletion, *ex.let* exitus letalis, *FCD* focal cortical dysplasia, *HME* hemimegalencephaly, *HET* heterotopia, *MTS* mesial temporal sclerosis, *N/A* not available, *PMG* polymicrogyria, *SWS* Sturge–Weber Syndrome, *f* female; *m* male, *L* left hemisphere, *R* right hemisphere

### DNA and RNA extraction

A prototypical area within the center of the MCD lesion (neocortex) was identified on H&E slides and macrodissection performed by punch biopsy (pfm medical, Köln, Germany) or by hand. DNA and RNA were extracted from formalin-fixed paraffin-embedded (FFPE) tissue using the Maxwell 16 FFPE Plus LEV DNA Kit and Maxwell 16 LEV RNA FFPE Purification Kit (Promega, Madison, WI, USA), according to the manufacturer’s instructions. DNA concentration was quantified using the Qubit dsDNA BR Assay kit (Invitrogen, Carlsbad, CA, USA).

### Genome-wide DNA methylation profiling and data pre-processing

Samples were analyzed using Illumina Infinium MethylationEPIC 850K BeadChip arrays, as described previously [16]. DNA methylation data were generated at the Department of Neuropathology, Universitätsklinikum Heidelberg, Germany. Copy number profile (CNP) analysis was assessed using R package “conumee” after an additional baseline correction (https://github.com/dstichel/conumee).

Differential DNA methylation analysis was performed with a self-customized Python wrapped cross R package pipeline, utilizing 'Champ,' 'watermelon,' 'DNAMarray,' and 'minfi' as R packages. 'MethQC' and 'Pymethylarray' were used as Python-based array analysis packages. Additional own implementations of lacking functionality were used where needed. All these packages were wrapped using 'rpy2′, an interface to R running embedded in a Python process. Methylation array data were read utilizing minfi’s ‘read_metharray_sheet’ and ‘read_metharray_exp’ function as well as a self-modified parallelized version available from the ‘DNAMarray’ R-package. Data were stratified quantile normalized using the ‘minfi’ ‘preprocessQuantile’ function. Probes targeting sex chromosomes, probes containing single nucleotide polymorphisms (SNPs) not uniquely matching, as well as known cross-reactive probes (see [18]) were removed. Additionally, a rpy2-wrapped and modified version of the ‘reduce’ and ‘probefiltering’ functions available in the ‘DNAMarray’ R-package were used for further processing of array data. Finally, 412,915 probes contained on the EPIC array were used for further analysis. Most significantly, differentially methylated CpGs between disease entities were identified by fitting a regression model with the disease as the target variable using the ‘limma’ R package. All pairwise comparisons between disease groups were identified as contrasts and included in the analysis. No surrogate variable adjustments (‘sva’ R package) or batch corrections were necessary. After identification of 96 unique most significantly differentially methylated CpGs (adj. *p* value < 0.01), unsupervised dimensionality reduction for cluster analysis was performed. Uniform Manifold Approximation and Projection (UMAP) for general non-linear dimensionality reduction was used for visualization [47]. After identification of disease clusters, care was taken that no cluster was confounded or correlated with any other variable such as sex, age at onset, age at surgery, duration of epilepsy, lobe (Supplement Fig. 1, online resource). Subsequently, additional hierarchical cluster analysis was performed.

### Machine and deep learning

‘Scikit-learn,’ ‘imbalanced-learn,’ ‘Keras,’ and ‘Tensorflow’ were used as Python-based packages to leverage machine and deep learning. The processed methylation data obtained were split into a training and independent test set. Care was taken that disease classes were stratified across the sets evenly. Afterwards, the training and test set were upsampled to retrieve sample groups of almost the same sizes using the Synthetic Minority Oversampling Technique (SMOTE) of the 'imbalanced-learn' package. Various 'classic' machine learning algorithms (using ‘Scikit-learn ‘) were spot-checked on their performance on the data via tenfold cross-validation (Supplement Fig. 1, online resource). Independently, sixfold stratified cross-validation was performed using a deep neuronal net architecture. The network consisted of four fully connected layers (one input and one output layer). The first three fully connected layers used a rectified linear activation function followed by a batch normalization layer and a subsequent dropout layer. Only the last layer had a sigmoid activation function. Neither batch normalization nor dropout did follow. The training was performed using a batch size of 64, cycling the learning rate between 0.00001 and 0.09 on every epoch for a total of 66 epochs, which was identified by early stopping. All parameters described were evaluated via a prior grid search. The network and training process was implemented using the Keras and Tensorflow Python-based deep learning frameworks.

### FISH and RNA sequencing

FISH analysis was performed on FFPE-sections from ten representative cases with and without a change in copy number profile. Sections from lesional (with PMG) and peri-lesional tissue blocks (no PMG) of the same patients were analyzed. Two color interphase FISH analysis was performed using 1q21 CKS1B Spectrum Orange / 1p32 CDKN2C Spectrum Green FISH Probe Kit (Vysis; Abbott). Pre-treatment of slides, hybridization, post-hybridization processing, and signal detection were performed as reported elsewhere [15]. Samples showing sufficient FISH efficiency (> 90% nuclei with signals) were evaluated by two independent investigators. Signals were scored in at least 300 non-overlapping, intact nuclei. Trisomy/gain of 1q was defined as > 15% of nuclei containing three or more signals for the respective locus probe, if no such findings were detected for the 1p locus (to rule out polyploidy).

RNA was obtained from the same samples and, upon reverse transcription, was subjected to next-generation sequencing on a NextSeq 500 (Illumina, San Diego, CA, USA), as described previously [63]. Four out of ten samples had low read numbers, but were still included in the analysis. We used deFuse [48] and Arriba (https://github.com/suhrig/arriba/) methods for the detection of gene fusions.

### Data Availability

Methylation data were deposited in NCBIs Gene Expression Omnibus (GEO, https://www.ncbi.nlm.nih.gov/geo; accession number GSE156374). Supplement Table 1, online resource, includes IDAT-file names for assignment to patient characteristics. The methylation data analysis pipeline used in this project is available on our project homepage (https://github.com/FAU-DLM/Methylr2py).

## Results

### Novel methylation cluster defines PMG among other MCD

**Fig. 1:**
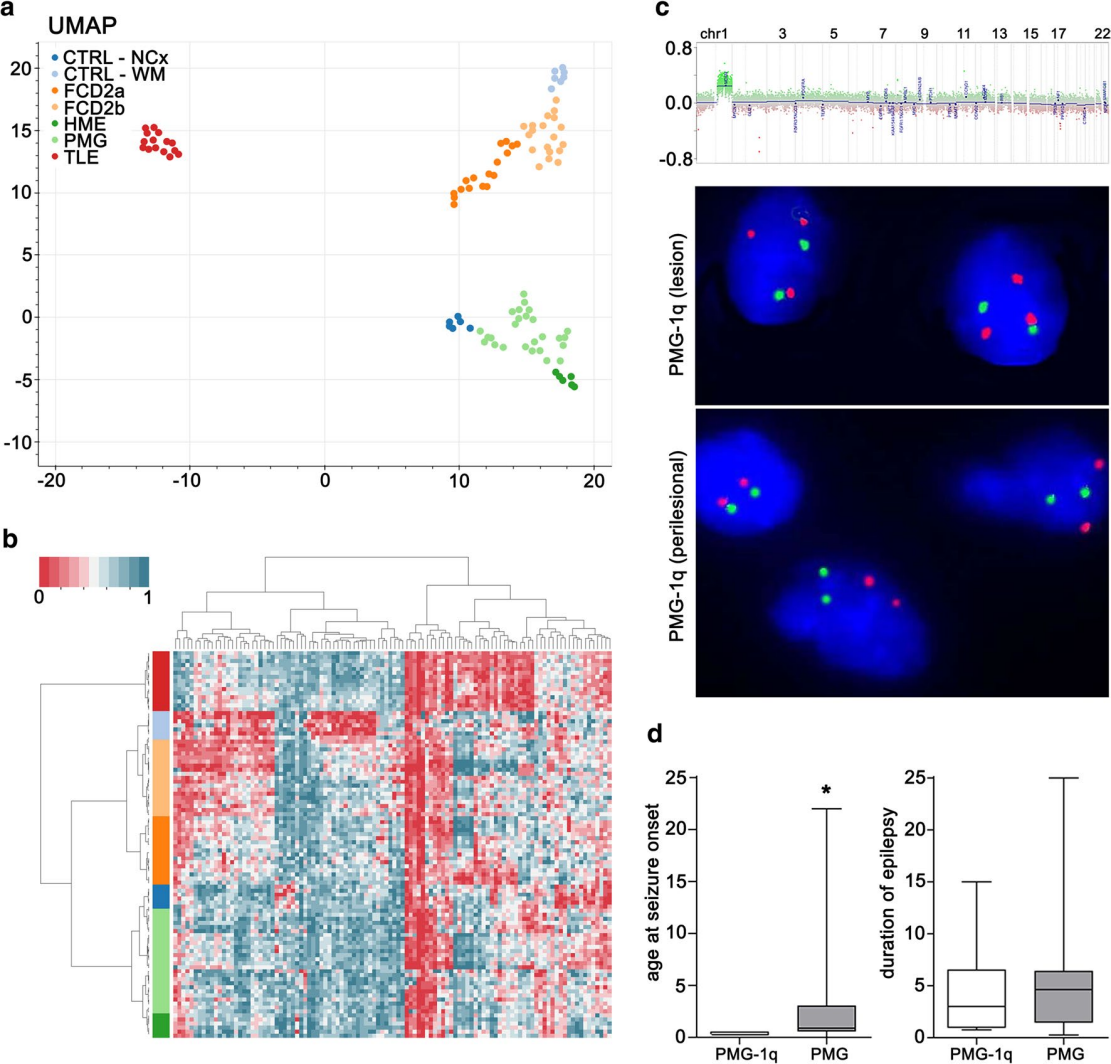
**a** UMAP and **b** hierarchical cluster analysis of PMG, reference MCD, non-MCD epilepsy, and no-seizure autopsy controls. Twenty-seven cases with histological diagnosis of PMG are indicated in light green. PMG cases formed a novel distinct methylation group. The color scheme for histopathological entities in (**a**) also applies to (**b**). **c** Copy number profiling analysis indicating duplication of chromosome arm 1q and FISH confirming brain somatic 1q triploid nuclei in the center of the lesion, but not in adjacent, architecturally normal-appearing tissue of the same patient. **d** 1q duplication in PMG patients associated with significantly earlier onset of seizures, but not longer duration of epilepsy before surgery. *Chr* chromosome, *CTRL* control, *NCx* neocortex, *WM* white matter, *FCD* focal cortical dysplasia, *HME* hemimegalencephaly, *PMG* polymicrogyria, *TLE* temporal lobe epilepsy, *UMAP* uniform manifold approximation and projection

To determine, whether DNA methylation signatures can be used to classify structurally related MCD molecularly, we used the methylation data from surgical brain samples obtained from 26 pharmacoresistant epilepsy patients with a histopathological diagnosis of PMG (Table 1) and 62 MCD and no-MCD reference cases (i.e., FCD type 2a, FCD type 2b, HME, TLE) as well as from no-seizure autopsy controls (CTRL, microdissected white matter and neocortex; Supplement Table 1, online resource). We performed unsupervised dimensionality reduction and hierarchical cluster analysis. In addition to previously described FCD- and TLE-specific methylation classes [38], PMG in our analysis formed a novel separate cluster in the UMAP dimensionality reduction (Fig. 1a). No confounding correlation with any other variable of our data was detected (e.g., sex, age, lobe; Supplement Fig. 1, online resource). Hierarchical cluster analysis confirmed the separation of samples at the disease level (Fig. 1b).

### Brain mosaic 1q triploidy as a new defining feature of some PMG

Next, we performed copy number profile analysis from DNA methylation data, which identified a uniform duplication spanning the entire long arm of chromosome 1 in 7/26 PMG cases (Fig. 1c). FISH analysis identified a consistent mosaic distribution of about 50% 1q triploid nuclei in the center of the PMG lesion of respective cases (mean ± SEM = 51.2% ± 1.3), but not in adjacent, architecturally normal-appearing tissue (mean ± SEM = 7.2% ± 3.0; Fig. 1c, lower panel). Comparison with PMG cases without 1q trisomy identified a significant positive correlation between the degree of mosaicism identified in FISH and the log_2_ copy number ratio identified with CNP analysis from DNA methylation array data (Pearson correlation, *R*^2^ = 0.99, *p* < 0.0001, *n* = 10; Supplement Table 2, online resource). Due to the retrospective study design, no blood samples were available from patients to confirm the mosaicism of the 1q duplication further. RNA-Seq analysis from same samples revealed the absence of translocations and gene fusions in all PMG patients with and without 1q copy number variation. Our findings provide evidence for a previously unrecognized PMG-specific methylation class and a brain mosaic chromosomal rearrangement affecting 1q in a subgroup of PMG patients.

### 1q trisomy associated with focal or hemispheric PMG, absence of hemimegalencephaly, early-onset severe focal epilepsy and developmental delay

**Fig. 2:**
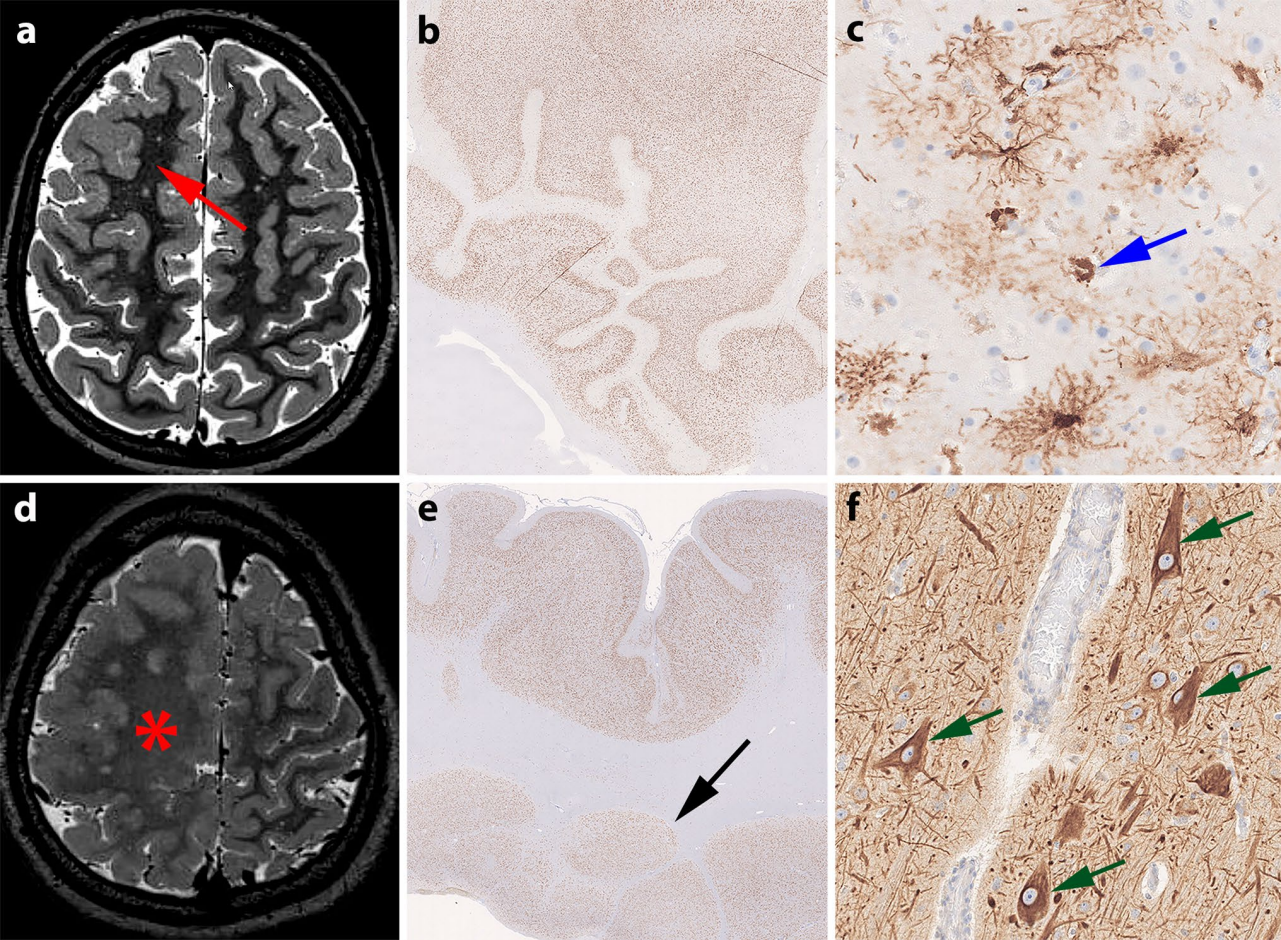
MRI and histology of representative PMG cases with and without brain mosaic 1q gain. **a** 15-year-old male patient (#6) with seizure onset at 3 months and an MRI-positive lesion in the right frontal lobe with cortical thickening and no hyperintense T2/FLAIR signal and no transmantle sign (arrow). **b** NeuN staining revealed abnormally folded sulci without pial opening. The cortical ribbon is small and mostly 4-layered. **c** GFAP-immunohistochemistry revealed protoplasmic astrocytic inclusions (blue arrow [28]). **d** 17-year-old male patient (#18) with seizure onset at 8 months and right hemimegalencephaly (asterisk). **e** Neocortical ribbon with small sulci without pial opening and nodular heterotopias in the white matter (black arrow; NeuN immunohistochemistry). **f** Dysmorphic neurons accumulating non-phosphorylated neurofilament protein (green arrows, SMI32 immunohistochemistry)

On MRI, PMG cases with 1q gain presented as unilateral focally restricted frontal or hemispheric PMG (Fig. 2a; Supplement Fig. 2, online resource). Histomorphological features of the PMG lesion included abnormally folded sulci without pial opening. The cortical ribbon was small and mostly only 4-layered (Fig. 2b). One case showed protoplasmic astrocytic inclusions (Fig. 2c, blue arrow). All 1q-positive PMG cases were clinically associated with a significantly earlier onset of seizures in the first few months of life (mean onset_PMG1q_ = 0.4 ± 0.04 years, *n* = 7) as compared to the other PMG in our cohort (mean onset_PMG_ = 2.3 ± 0.7 years, *n* = 19, Mann–Whitney test, *p* = 0.018; Fig. 1d). The mean duration of epilepsy before surgery was not different between the two molecular PMG subgroups (mean duration_PMG1q_ = 4.3 ± 1.9 years, *n* = 7; mean duration_PMG_ = 5.5 ± 1.4 years, *n* = 19; Mann–Whitney test, *p* = 0.48; Fig. 1d). Based on the available routine clinical reports, there was no evidence for a specific seizure pattern that would distinguish this patient group. Six out of seven PMG patients with brain mosaic 1q duplication presented with a severe combinatorial developmental delay including cognition, speech, and motor. Five out of seven patients had a post-surgical outcome of Engel class 1 5 years after surgery [19]. Two patients did not become seizure free due to incomplete resection of the epileptogenic zone to avoid hemiparesis.

In contrast, PMG cases without 1q duplication showed greater variability in their clinical presentation. In addition to the significantly later epilepsy onset, these cases more frequently presented with their PMG as part of a hemimegalencephaly (Fig. 2d, red asterisk; Supplement Fig. 3, online resource) or otherwise complex cortical malformation with, e.g., cortical thickening, broad signal alterations in the white matter, blurring of the gray–white matter junction, periventricular or subcortical nodular heterotopias (Fig. 2e, black arrow), and FCD type 2 (Fig. 2f, green arrows), schizencephaly or microcephaly (Table 1). Two patients in this subgroup were diagnosed with Sturge–Weber Syndrome. Five patients had no or only mild-to-moderate intellectual disability and developmental delay. Thirteen patients were reported with severe combinatorial developmental delay. For one case, we had no detailed information. One patient had a reported 22q11 microdeletion. Another one had a reported congenital cytomegalovirus infection. However, no systematic genetic or viral testing was performed in the cohort. Thirteen out of 19 patients became seizure free in this subgroup.

## Discussion

A growing list of PMG types and syndromes have been observed, most of which have not been completely delineated. Their description is primarily based on their localization (by MRI) and correlation with clinical aspects including developmental course, growth anomalies, and dysmorphism, seizure history, family history, and genetic testing of blood for PMG-associated genes [68]. Here, we studied a series of 26 patients with pharmacoresistant epilepsy who underwent surgical treatment and were diagnosed with a PMG based on imaging and histopathology. From this cohort, we extracted a set of 7 patients exhibiting a unique molecular fingerprint along with specific clinical features including early-onset epilepsy in the first months of life and severe combinatorial developmental delay, thereby defining a distinct PMG entity. The molecular fingerprint relied on both a highly characteristic methylation profile and invariable detection of a brain mosaic duplication of the long arm of chromosome 1.

MCD are the most frequent causes of focal childhood epilepsies, carrying a lifelong disability perspective and reduced quality of life [12]. They represent a wide range of cortical lesions resulting from derangements of normal intrauterine developmental processes and involving cells implicated in the formation of the cortical mantle [61]. The pathological features depend mainly on the timing of the defect in the developmental processes and the cause, e.g., abnormal proliferation or apoptosis, differentiation, neuronal migration, or layering [66]. Classification of MCD has proven challenging as two or more forms of MCD may coexist. Also, a particular defect in corticogenesis may give rise to more than one morphological subcategory of MCD, and, conversely, a morphological subtype of MCD may have more than one mechanism for its formation [3]. Thus, there is a need to complement current classification of MCD with molecular-genetic data.

The impact of integrating molecular genotypes with histopathological phenotypes for the classification of brain tumors was emphasized by the WHO in 2016 [46]. Beyond disease-related genetic variants, genomic DNA methylation classifiers have been identified as a valuable source in the decision-making process for disease diagnosis, prognosis, and treatment especially in brain tumors and other cancers [16]. In addition, we were recently able to show that genomic DNA methylation signatures also distinguished major FCD subtypes from TLE patients and non-epileptic controls [38]. Now, we confirmed and extended this finding in a new independent patient cohort, including FCD among other MCD (i.e., PMG, HME), TLE, and no-seizure autopsy controls. All disease groups were identified to fall into distinct methylation classes, as shown by unsupervised dimensionality reduction and hierarchical cluster analysis. At the point of writing this manuscript, there had been no previous report on PMG-specific DNA methylation signatures.

Performing copy number profiling from DNA methylation data, we next identified a somatic duplication of the entire long arm of chromosome 1 in surgical brain tissue of a subset of PMG patients. Chromosome 1 is the largest human chromosome, with a length of 248,956,422 bp and 2055 coding and 2092 non-coding genes (ENSEMBL, GRCh38.p13). It is highly susceptible to genetic variations such as polymorphisms or mutations, and many diseases have been linked to these abnormalities. Complete monosomy is invariably lethal before birth and complete trisomy is lethal within days after conception [6]. Symptoms of congenital partial deletions and partial duplications generally depend on the size and location of the anomaly, and the genes involved. Several 1q related microdeletion and duplication syndromes or translocations are known. Features that may occur in respective patients include developmental delay and learning disabilities, slow growth and short stature, various congenital disabilities (such as cleft palate or a heart defect), specific facial features (such as a small, receding jaw), as well as neoplasias. This manifold of diseases highlights the central role of genes on the long arm of chromosome 1 in overall development, but also brain structure and function. For example, recurrent rearrangements of 1q21.1 are associated with microcephaly or macrocephaly, developmental, behavioral, and psychiatric problems (e.g., autism spectrum disorders, attention-deficit disorder, learning disabilities, schizophrenia), and seizures [14, 25, 49, 53]. 1q24 deletions cause a phenotype of intellectual disability, growth retardation, microcephaly, and facial dysmorphism [17]. Patients with 1q41 microdeletion syndrome were reported to present with seizures, mental retardation or developmental delay, and dysmorphic features at varying degrees [60]. Another microdeletion affecting 1q43q44 was associated with corpus callosum abnormalities, microcephaly, intellectual disability, and seizures [13, 21, 30, 45]. Of the large variety of germline mutations that have been previously associated with PMG, two mapped to chromosome 1q: *AKT3* (chr1q43-44) and *FH* (chr1q43)*,* [20, 27, 55]. Of all CNVs that have been described in PMG [23, 57], none mapped to 1q before. To the best of our knowledge, the present study provides the first description of a 1q duplication syndrome affecting the entire long arm of chromosome 1 in focal epilepsy patients with a histopathological diagnosis of PMG. Confirmatory FISH further identified a consistent mosaic distribution of 1q triploid nuclei in the center of the PMG lesion, but not in peri-lesional normal-appearing tissue. So far, somatic variants in focal epilepsy patients with an underlying MCD have been identified only in FCD type 2 and HME [2, 4, 5, 20, 44, 50–52]. There had been no previous descriptions of brain somatic variants in PMG.

Differential DNA methylation in PMG cases with 1q trisomy affected not only regions on the long arm of chromosome 1, but also was seen in other autosomes and mapped to several genes associated with brain function, malformation, epilepsy, developmental delay, and autism (Supplement Fig. 4, online source) [1, 22, 24, 29, 31, 32, 40, 56, 64, 67]. Our observation suggests, that the DNA methylation signature did not only result from the 1q duplication, but represented a complex pattern including the development of the structural lesion, chronic seizures, and co-morbidities [11, 36, 37, 41]. A potentially causative role of DNA methylation changes in the development of cortical malformations and associated seizures may also be envisaged. A much higher genomic coverage and high throughput sequencing methods will be needed to address this issue, in particular in epilepsy samples, where we recently detected the majority of disease- and pathology-associated DNA methylation changes outside of regions covered by Illumina tiling arrays [38].

Our results are in line with a recently published international consensus recommendation on the diagnostic work-up for MCD [59]. This expert panel recognized PMG as one of the most frequent and most heterogeneous malformations in etiology with currently only 9–20% of cases yielding a (blood-based) molecular diagnosis. Although surgical brain tissue is not always available for the clinical work-up of MCD patients, our study showed that identification of brain mosaicism and DNA methylation analysis from histopathologically well-characterized tissue can help to increase the diagnostic yield and further sharpen the phenotypic and genotypic spectrum of MCD, in particular for the heterogeneous group of PMG. Hence, molecular diagnosis is endorsed in the diagnostic workflow to maximize the diagnostic yield in MCD per se and hopefully to increase the number of patients receiving personalized care and counseling on prognosis and recurrence risk.

## Conclusion

We describe the invariable coincidence of a specific methylation profile with the presence of a brain somatic trisomy involving chromosome 1q in a distinct group of PMG patients without hemimegalencephaly, with early-onset epilepsy in the first months of life and severe developmental delay. Other PMG showed no chromosomal imbalances, formed a distinct methylation class in hierarchical cluster analysis, more often presented as complex malformation on MRI and histologically, and showed considerable higher variability of clinical phenotype. Seventeen PMG patients in the present study, 5 with 1q duplication and 13 without, had a post-surgical outcome of Engel class 1 five years after surgery. Thus, irrespective of the small study cohort, our data suggest that surgical treatment is recommended in pharmacoresistant focal epilepsy patients with unilateral PMG and associated with favorable seizure outcome. Our experimental data support an integrated molecular-genetic and histological disease classification of cortical malformations including PMG. Moreover, we identify a new patient group with unilateral PMG, very early-onset focal epilepsy and brain mosaic 1q whole-arm duplication.

## Electronic supplementary material

Below is the link to the electronic supplementary material.

Supplementary file1 (PDF 1226 kb)
