# Supplementary material for: Mosaic trisomy of chromosome 1q in human brain tissue associates with unilateral polymicrogyria, very early-onset focal epilepsy, and severe developmental delay
Source: Acta Neuropathol. 2020 Sep 26;140(6):881–91. doi: 10.1007/s00401-020-02228-5 (PMC7666281; doi:10.1007/s00401-020-02228-5)
Supplement: Supplementary file 1 — Supplementary file1 (PDF 1226 kb) [file 401_2020_2228_MOESM1_ESM.pdf]

Supplement Figure 1

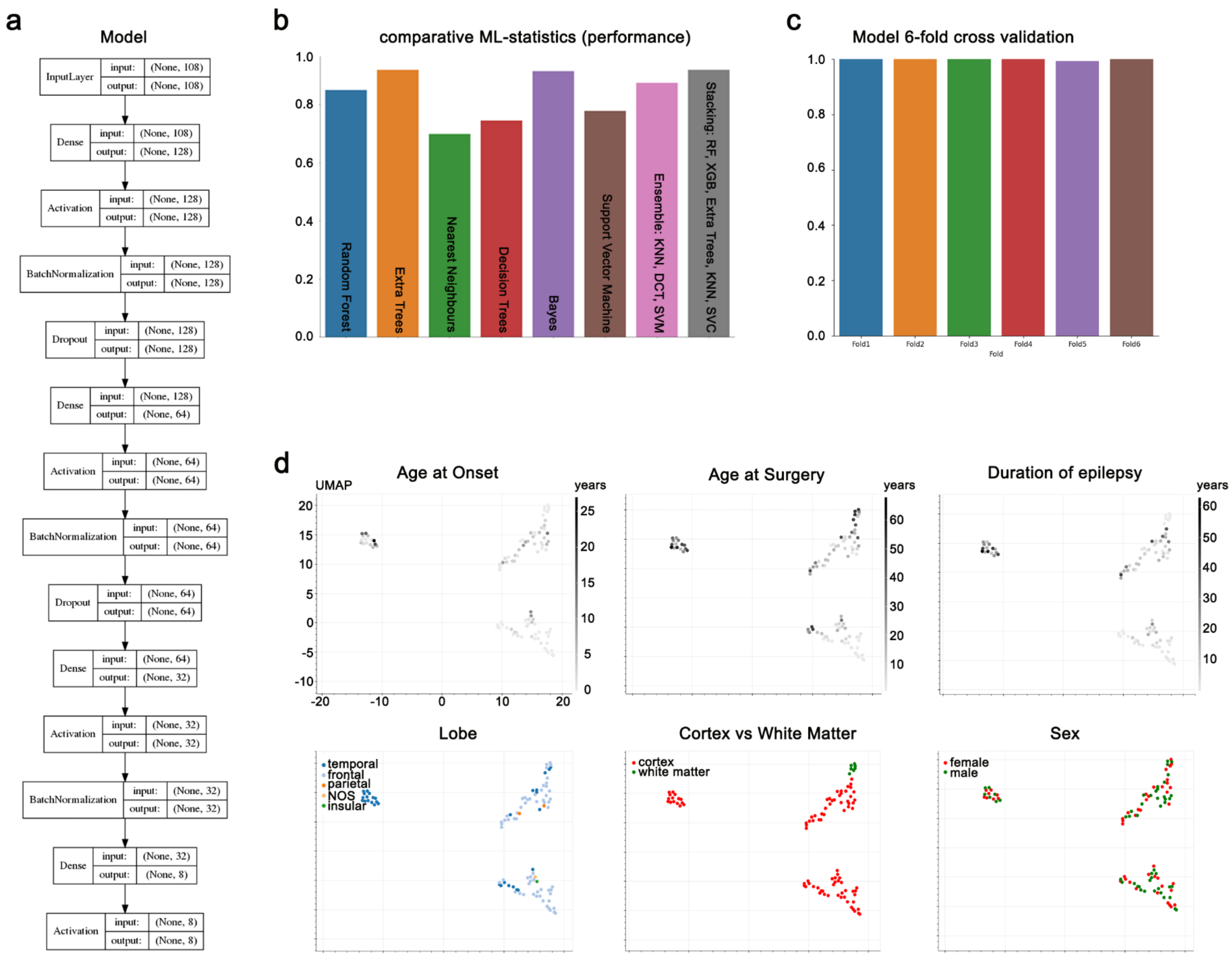

Legend to Supplement Fig. 1: (a) A neural network was trained for DNA methylation-based disease classification. **(b-c)** Comparison with other machine learning methods showed superior performance of the neural network in 6-fold cross-validation. **(d)** UMAP plots were generated from 108 most significantly differentially methylated positions with potentially confounding variable influence. Samples were labelled with the covariates age at onset, age at surgery, duration of epilepsy, sampled lobe (temporal, frontal, parietal, insular, NOS – not otherwise specified), cortex (green) or white matter (red), and sex, male (green), female (red). Clustering of samples was not driven by the tested covariates.

Supplement Figure 2

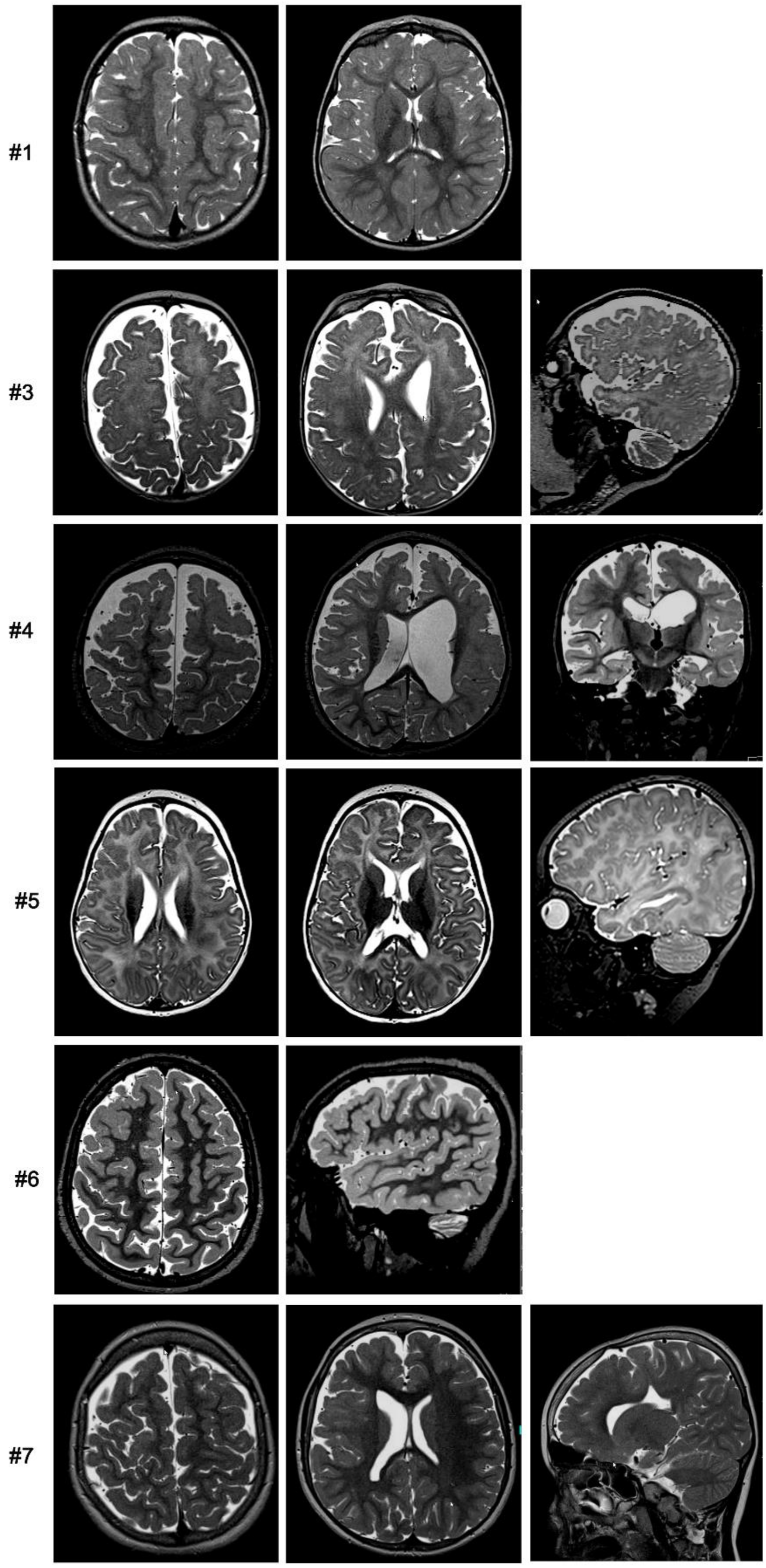

Legend to Supplement Figure 2: Representative MRIs of PMG-1q patients. No HME was present in this patient subgroup.

Supplement Figure 3

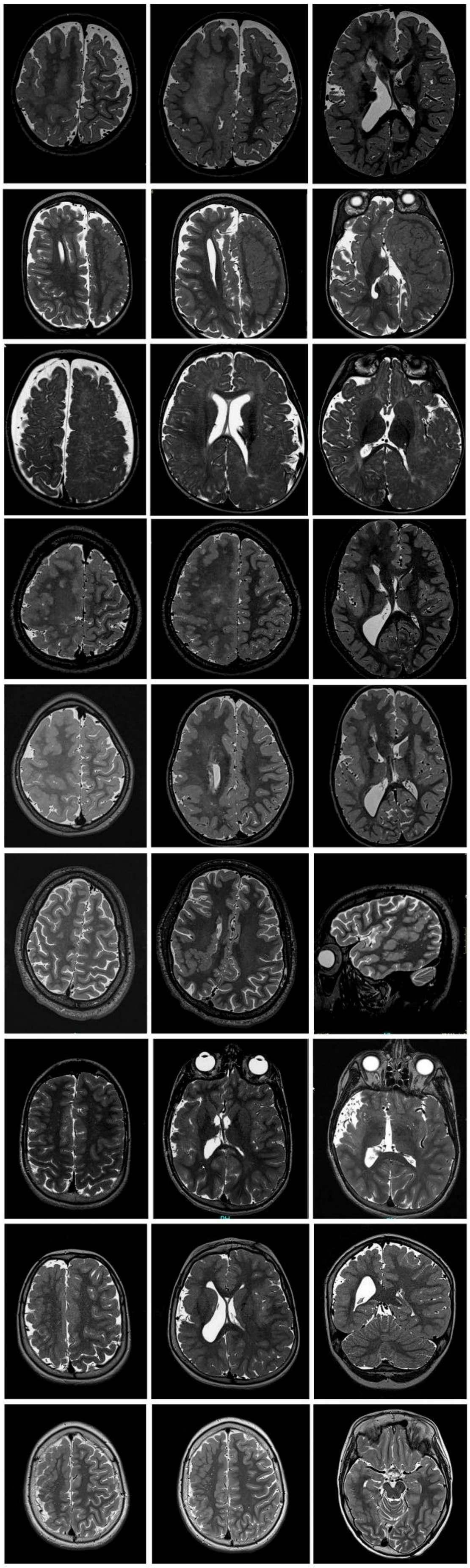

#9

#13

#16

#18

#19

#20

#22

#23

#24

Legend to Supplement Figure 3: Representative MRI of PMG patients without 1q duplication. In these cases, PMG was more frequently part of HME or other complex malformations.

Supplement Figure 4

a

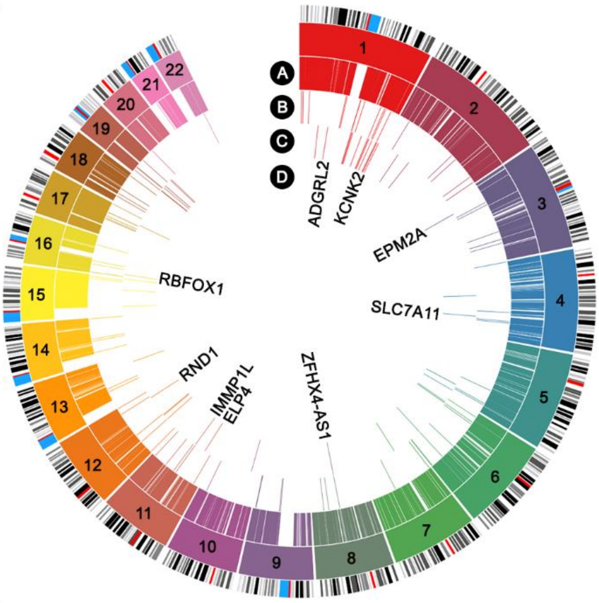

b

Gene	Chr	Relevance	References
ADGRL2	1	microcephaly	Vezain et al. 2018
ELP4	11	epilepsy, mental retard., autism	Strug et al. 2009; Addis et al. 2015
EPM2A	3	epilepsy	Ganesh et al. 2002
IMMP1L	11	mental retardation	Hu et al. 2015
KCNK2	1	epilepsy	Heurteaux et al. 2004; Djillani et al. 2019
RBFOX1	16	epilepsy	Lal et al. 2015
RND1	12	brain development and function	Ishikawa et al. 2003
SLC7A11	4	seizures	Robert et al. 2015
ZFHX4-AS1	8	brain specific lncRNA	

Legend to Supplement Figure 4: (**a**) Circos plot created with Circa (http://omgenomics.com/circa) showing on outer rings human chromosomes with cytobands (hg19). Inner rings summarize genomic mapping of DMPs (adj.p<0.01) A) identified in all pairwise comparisons, B) used in UMAP and cluster analysis, as well as C) identified specifically from pairwise comparison between PMG with and without 1q trisomy and D) gene names. Differential methylation signals demonstrated an even distribution across all autosomes, with no evidence for clustering at particular chromosomes or gross chromosomal regions (e.g., towards central autosome domains or ends). (**b**) DMP mapping to genes provided evidence for functional associations with brain development, brain malformation, epilepsy, mental retardation, and autism.

ADGRL2 - Adhesion G Protein-Coupled Receptor L2; *ELP4* - Elongator Acetyltransferase Complex Subunit 4; *EPM2A* – Epilepsy, Progressive Myoclonus Type 2A Glucan Phosphatase, Laforin; *IMMP1L* - Inner Mitochondrial Membrane Peptidase Subunit 1; *KCNK2* - Potassium Two Pore

Domain Channel Subfamily K Member 2; *RBFOX1* - RNA Binding Fox-1 Homolog 1; *RND1* - Rho Family GTPase 1; *SLC7A11* - Solute Carrier Family 7 Member 11; *ZFHX4-AS1* - Zinc Finger Homeobox 4 Antisense RNA 1; Chr – chromosome; lncRNA – long non-coding RNA.

Supplement Table 1: Clinical summary of reference cohort and idat allocation.

#	pathology	lobe	sex	age_onset	duration	age_surgery	idat
1	PMG	frontal	F	0	3	3	203220070058_R06C01
2	PMG	frontal	M	0	1	1	203220070058_R07C01
3	PMG	frontal	M	0	1	1	203219750116_R02C01
4	PMG	frontal	M	0	3	3	203219750116_R04C01
5	PMG	frontal	F	0	1	1	203219730055_R03C01
6	PMG	NOS	F	0	15	15	203219730055_R06C01
7	PMG	frontal	F	0	7	7	203511880025_R05C01
8	PMG	temporal	M	2	6	8	203220070058_R05C01
9	PMG	frontal	M	0	3	3	203219750116_R08C01
10	PMG	frontal	M	1	1	2	203219730055_R04C01
11	PMG	temporal	F	11	6	17	203219730055_R05C01
12	PMG	frontal	M	0	6	6	203511880014_R08C01
13	PMG	frontal	M	1	2	3	203511880025_R01C01
14	PMG	frontal	F	0	1	2	203511880025_R02C01
15	PMG	frontal	F	0	2	2	203511880025_R03C01
16	PMG	frontal	M	0	1	1	203511880025_R04C01
17	PMG	frontal	M	1	7	8	203511880025_R06C01
18	PMG	frontal	M	1	16	17	203219750146_R05C01
19	PMG	frontal	F	0	5	5	203511880026_R02C01
20	PMG	frontal	M	10	25	35	203511880026_R03C01
21	PMG	temporal	M	3	2	5	203220070058_R04C01
22	PMG	temporal	M	3	10	13	203220070058_R08C01
23	PMG	frontal	M	2	5	7	203219750116_R03C01
24	PMG	Insel	M	3	6	9	203219750116_R05C01
25	PMG	frontal	F	4	4	8	203219750116_R06C01
26	PMG	frontal	M	0	2	2	203219750116_R07C01
27	CTRL - NCx	temporal	F			11	202818860053_R04C01
28	CTRL - NCx	temporal	M			27	202818860053_R06C01
29	CTRL - NCx	frontal	M			27	202818860053_R08C01
30	CTRL - NCx	temporal	F			13	202931510124_R04C01
31	CTRL - NCx	temporal	F			49	202931510124_R06C01
32	CTRL - NCx	frontal	F			49	202931510124_R08C01
33	CTRL - WM	temporal	F			11	202818860053_R05C01
34	CTRL - WM	frontal	M			27	202931510124_R01C01
35	CTRL - WM	temporal	F			13	202931510124_R05C01
36	CTRL - WM	temporal	F			49	202931510124_R07C01
37	CTRL - WM	frontal	F			49	202939390010_R06C01
38	CTRL - WM	frontal	M			52	202939390010_R08C01
39	CTRL - WM	frontal	M			52	202944920003_R06C01
40	FCD 2A	frontal	M	2	2	4	202818860117_R02C01
41	FCD 2A	frontal	M	0	1	1	202818860117_R06C01
42	FCD 2A	frontal	F	0	3	3	202827620174_R03C01
43	FCD 2A	frontal	F	8	0	8	202827620174_R04C01
44	FCD 2A	frontal	F	1	9	9	202093110113_R03C01
45	FCD 2A	frontal	M	4	9	13	202822930036_R07C01
46	FCD 2A	frontal	F	3	7	10	202818860117_R05C01
47	FCD 2A	temporal	M	0	6	6	202827620173_R08C01
48	FCD 2A	frontal	F	10	8	18	202827620174_R01C01
49	FCD 2A	frontal	M	5	6	11	202827620174_R06C01
50	FCD 2A	frontal	M	3	14	17	202093110108_R07C01
51	FCD 2A	frontal	M	9	15	24	202093110113_R01C01
52	FCD 2A	frontal	F	0	19	19	202093110113_R02C01
53	FCD 2A	frontal	F	4	11	15	202818860117_R01C01
54	FCD 2A	parietal	F	1	11	12	202818860117_R04C01
55	FCD 2A	frontal	F	0	45	45	202093110108_R08C01
56	FCD 2A	frontal	F	2	30	32	202822930036_R06C01
57	FCD 2B	frontal	M	1	1	2	202831040055_R05C01
58	FCD 2B	frontal	F	0	3	3	202831040055_R08C01
59	FCD 2B	parietal	M	0	11	11	202831040056_R01C01
60	FCD 2B	frontal	M	6	2	8	202831040056_R06C01
61	FCD 2B	frontal	M	2	5	7	202831040056_R08C01
62	FCD 2B	temporal	M	0	5	5	202822930161_R03C01

63	FCD 2B	frontal	M	4	2	6	202822930161_R05C01
64	FCD 2B	temporal	M	0	5	5	202822930161_R06C01
65	FCD 2B	frontal	F	3	7	10	202831040055_R01C01
66	FCD 2B	frontal	M	1	7	8	202831040056_R04C01
67	FCD 2B	frontal	F	1	15	16	202831040055_R03C01
68	FCD 2B	frontal	F	4	11	15	202831040055_R07C01
69	FCD 2B	temporal	M	1	15	16	202831040056_R05C01
70	FCD 2B	frontal	F	5	15	20	202831040056_R07C01
71	FCD 2B	frontal	M	5	15	20	202822930161_R02C01
72	FCD 2B	frontal	F	13	30	43	202148010052_R02C01
73	FCD 2B	frontal	M	3	40	43	202831040055_R04C01
74	FCD 2B	frontal	M	2	36	38	202831040056_R03C01
75	FCD 2B	temporal	F	5	41	46	202822930161_R04C01
76	HME	frontal	M	0	1	1	202827620174_R07C01
77	HME	frontal	M	1	1	1	202827620174_R08C01
78	HME	frontal	F	0	2	2	203219750146_R04C01
79	HME	frontal	F	0	1	1	203219750146_R06C01
80	HME	frontal	F	0	2	2	203219750057_R01C01
81	HME	frontal	F	0	1	1	203219750057_R02C01
82	TLE	temporal	M	16	10	26	202148010059_R02C01
83	TLE	temporal	F	2	17	19	202148010053_R08C01
84	TLE	temporal	F	27	16	43	202148010058_R02C01
85	TLE	temporal	F	4	11	15	202148010058_R03C01
86	TLE	temporal	F	1	15	15	202148010058_R06C01
87	TLE	temporal	M	6	23	29	202148010053_R06C01
88	TLE	temporal	F	18	23	41	202148010053_R07C01
89	TLE	temporal	M	6	44	50	202148010058_R01C01
90	TLE	temporal	F	3	25	28	202148010058_R04C01
91	TLE	temporal	M	1	44	45	202148010058_R07C01
92	TLE	temporal	M	15	25	40	202148010059_R01C01
93	TLE	temporal	F	5	28	33	202148010059_R03C01
94	TLE	temporal	M	1	47	48	202148010059_R04C01
95	TLE	temporal	M	1	54	55	202148010058_R05C01
96	TLE	temporal	F	5	63	68	202148010058_R08C01

Supplement Table 2: Quantification of 1q mosaicism from FISH and CNP.

#	Diploid nuclei	Nuclei with 1q trisomy	1q ratio in 850k
2	52%	48%	0.35
3	48%	52%	0.35
4	44%	56%	0.35
5	46%	54%	0.34
6	51%	49%	0.34
8	88%	12%	0.00
9	92%	8%	0.00
12	87%	13%	0.00
18	85%	15%	0.00
24	88%	12%	0.00
